# *In vitro* immunogenic profile of recombinant SARS-CoV2 S1-RBD peptide in murine macrophage and microglial cells

**DOI:** 10.1590/0074-02760220144

**Published:** 2023-03-31

**Authors:** Adriano José Maia Chaves, Paloma Marinho Jucá, Michelle Verde Ramo Soares, Caio Andrade de Oliveira, Raul Cavalcante de Sousa, Deniele Bezerra Lós, Remo Castro Russo, Juliana Navarro Ueda Yaochite, Danielle S Macedo

**Affiliations:** 1 Universidade Federal do Ceará, Faculdade de Medicina, Departamento de Fisiologia e Farmacologia, Núcleo de Pesquisa e Desenvolvimento de Medicamentos, Laboratório de Neurofarmacologia, Fortaleza, CE, Brasil; 2 Universidade Federal de Minas Gerais, Instituto de Ciências Biológicas, Departamento de Fisiologia e Biofísica, Laboratório de Imunologia e Mecânica Pulmonar, Belo Horizonte, MG, Brasil; 3 Universidade Federal do Ceará, Faculdade de Farmácia, Odontologia e Enfermagem, Departamento de Análises Clínicas, Fortaleza, CE, Brasil

**Keywords:** SARS-CoV2 S1 receptor-binding domain (RBD), macrophage, microglia, neuroinflammation, innate immunity

## Abstract

**BACKGROUND:**

The novel severe acute respiratory syndrome coronavirus 2 (SARS-CoV-2) variants can infect common mice inducing significant pathological lung lesions and inflammatory responses. This substantially mimics coronavirus disease 19 (COVID-19) infection and pathogenesis in humans.

**OBJECTIVES:**

To characterise the effects of recombinant SARS-CoV-2 S1 receptor-binding domain (RBD) peptide in murine macrophage and microglial cells’ immune activation compared with classical PAMPs *in vitro*.

**METHODS:**

Murine RAW 264.7 macrophages and BV2 microglial cells were exposed to increasing concentrations of the RBD peptide (0.01, 0.05, and 0.1 µg/mL), Lipopolysaccharide (LPS) and Poly(I:C) and evaluated after two and 24 h for significant markers of macrophage activation. We determined the effects of RBD peptide on cell viability, cleaved caspase 3 expressions, and nuclear morphometry analysis.

**FINDINGS:**

In RAW cells, RBD peptide was cytotoxic, but not for BV2 cells. RAW cells presented increased arginase activity and IL-10 production; however, BV2 cells expressed iNOS and IL-6 after RBD peptide exposure. In addition, RAW cells increased cleaved-caspase-3, apoptosis, and mitotic catastrophe after RBD peptide stimulation but not BV2 cells.

**CONCLUSION:**

RBD peptide exposure has different effects depending on the cell line, exposure time, and concentration. This study brings new evidence about the immunogenic profile of RBD in macrophage and microglial cells, advancing the understanding of SARS-Cov2 immuno- and neuropathology.

Myeloid cells, including monocytes and macrophages, are the subpopulations of immune cells that have pivotal functions in the immunity and host defence against various body-invasive pathogens, including those resident populations belonging to the barrier tissue from mucosal sites, such as the skin and lungs.^1^ Macrophages contribute to an extensive range of immune functions, such as antigen presentation, T-cell activation, and production/secretion of cytokines and chemokines.^2^ Despite this, macrophages and other antigen-presenting cells usually represent the first defence line of innate immunity modulating the subsequent adaptive immune response.^1^ ^,^ ^2^ However, recent evidence has demonstrated abnormalities in monocytes/macrophages’ function and phenotype in blood and post-mortem samples of coronavirus disease 19 (COVID-19) patients.^3^ ^,^ ^4^ Based on this, the role of these cells has been changing from beneficial arms of innate immunity to a potential branch of initiators and maintainers of inflammation and cytokine storm.^5^ ^,^ ^6^ ^,^ ^7^

The emergence of severe acute respiratory syndrome (SARS) induced by the COVID-19 spread rapidly. It became a worldwide pandemic, overtaking 505.817.953 confirmed cases of infection, achieving 6.213.876 deaths, reported to World Health Organization (WHO) until April of 2022 (https://covid19.who.int/). Severe acute respiratory syndrome coronavirus 2 (SARS-CoV-2) is a virus in the Betacoronavirus genus. This positive-sense single-stranded RNA virus exhibits 80% of similarities to SARS-CoV in genome structure, resembling the manifestations of tissue tropism and viral pathogenesis.^8^ SARS-CoV-2 is highly transmissible with a broad tissue tropism, entering the host via the respiratory tract, with subsequent airway spread, initially infecting the bronchial and alveolar epithelial cells, leading to acute lung injury, cytokine storm, and tissue failure.^8^ However, the vascular endothelial cells and alveolar macrophages are also important targets of viral infection and activation, contributing to higher proportions of pro-inflammatory cytokine sources and severe symptoms of COVID-19.^8^ Besides respiratory manifestations, SARS-CoV-2 infection induces neurological and neuropsychiatric symptoms, such as ageusia, anosmia, headaches, dysphoria, delirium, and mood disorders.^9^ ^,^ ^10^ In addition, the virus may reach the brain through the olfactory-hematogenous, trans-neuronal, and lymphatic pathways.^11^

Additionally, part of SARS-CoV2-induced central nervous system (CNS) alterations can also be attributed to the systemic inflammatory response caused by the virus and subsequent neuroinflammation.^12^ Microglia are brain-resident macrophages that patrol their surrounding microenvironment by projecting and retracting their highly motile processes. Hence, in the presence of injury or pathogens within the CNS, these cells become activated (with retracted processes), being capable of producing high amounts of pro-inflammatory cytokines, nitric oxide (NO), and radical oxygen species (ROS).^13^ ^,^ ^14^

The SARS-CoV-2 spike (S) protein is one of the most potent antigenic proteins during current viral infection.^15^ This protein is composed of two subunits, S1 and S2. The S1 subunit harbours the receptor-binding domain (RBD), which mediates the virus entry in host cells through its interaction with angiotensin-converting enzyme 2 (ACE2).^16^ Immunohistochemistry demonstrated high expressions of ACE2 on human tissue macrophages, such as alveolar macrophages and microglial cells in the brain, at a steady state.^17^ Recent evidence shows that RBD of SARS-CoV-2 (B.1.351) S protein turned to a high binding affinity to mouse angiotensin-converting enzyme 2 (mACE2), turning the mice highly susceptible to SARS-CoV-2 (B.1.351) infection.^18^ Besides this, recent studies have described ACE2’s independent pro-inflammatory properties of S protein since it also interacts with pattern recognition receptors (PRR), activating an innate immune response. In line with this, Zhao et al.^19^ demonstrated that S protein activates the PRR, toll-like receptor (TLR) 4, in human macrophages, promoting MyD88-dependent pro-inflammatory cytokine expression.^19^ Also, the S protein can activate human microglia cells disrupting mitochondria biogenesis and O2 consumption.^20^ In the BV2 cell line, the S1 subunit’s ability to induce a marked pro-inflammatory activation is blocked by TLR4 pharmacological and small interfering RNA inhibition.^21^ As previously mentioned, wild-type mice can be infected by SARS-CoV-2 B.1.351 variant indicating a possible novel cross-species transmission route.^18^ This evidence turns murine cells into an essential tool for investigating the pathogenesis and evaluating antiviral inhibitors and vaccines.

Due to the high immunogenic properties of RBD, it is one of the preferred targets for efficient immunisation strategies.^22^ Nevertheless, little is known about the specific role of RBD in activating innate immune cells relevant to the systemic and local (brain) immune response mounted by the SARS-CoV-2, nor the influence of this peptide’s concentrations in this effect. Therefore, the present study aimed to characterise the time- and concentration-dependent alterations induced by RBD exposure in murine macrophage and microglial cell lines. Furthermore, we hypothesised that the RBD at higher concentrations could induce a pro-inflammatory macrophage and microglia profile not or less observed at lower concentrations.

## MATERIALS AND METHODS

*Drugs and RBD obtention* - Dulbecco’s Modified Eagle’s Medium (DMEM), penicillin-streptomycin solution, glutamine, and foetal bovine serum (FBS) were obtained from GIBCO Laboratories (NY, USA). Recombinant SARS-CoV-2 S1 RBD peptide from SARS-CoV-2 B1 lineage was purchased from BioLinker (São Paulo, Brazil). For RBD production, the clones of the genes were obtained in the expression vector pET28a by chemical synthesis from Twist Bioscience (USA). RBD was produced in the *Escherichia coli* (BL21 cells) expression system. The purification was performed by His-Tag, while purity was determined by HPLC and SDS-PAGE electrophoresis (> 95%).^23^ The recombinant protein was tested for endotoxin presence and was approved for usage if < 1.0 EU/µg of endotoxin protein. Lipopolysaccharide (LPS) from *E. coli*, strain 055:B5, and Polyinosinic:Polycytidylic acid sodium salt [Poly(I:C)] was purchased from Sigma-Aldrich (Corp., St Louis, USA). The antibodies against inducible nitric oxide synthase - iNOS (catalog #13120) and cleaved (Asp175)-caspase-3 (catalog #117397) were purchased from Cell Signaling Tech (Massachusetts, USA) and Thermo-Fisher Scientific (California, USA), respectively.

*Cell culture and ethical aspects* - The cell lines used in the present study, *i.e.*, murine RAW 264.7 macrophages and murine BV2 microglia, do not require ethical approval in basic research. Both cell lines were purchased from the Banco de Células do Rio de Janeiro (RAW 264.7 code 0212, RAW 264.7 | APABCAM (bcrj.org.br; BV2 code 0356 BV-2 | APABCAM (bcrj.org.br)). The cells were grown in DMEM supplemented with 10% heat-inactivated FBS, 1% glutamine, and antibiotics (100 U/mL penicillin, 100 U/mL streptomycin, HVD Life Sciences) under 95% relative humidity with 5% CO2 at 37ºC. We renewed and maintained the cell stock at -80ºC. Experiments were performed in exponential growing cells, never exceeding P20. Cells tested negatively for mycoplasma at the beginning of the experimental procedures. Cells were initially seeded in T25 cm2 plastic bottles until they reached a confluence of approximately 80%. Then, they were submitted to mild trypsinisation (0.05% trypsin-EDTA for 05 min at 37ºC) and reseeded according to the following experimental protocol.

*Experimental design* - To assess the time-dependent immune activation profile of RBD in RAW 264.7 macrophages and BV2 microglial cells, we divided the experiments into two independent time points set at 2 and 24 h after antigen exposure. The experiment consisted of cells exposed to RBD peptides at the concentrations of 0.01, 0.05, and 0.1 µg/mL (groups named RBD0.01, RBD0.05, and RBD0.1), LPS at 1, and 5 µg/mL (groups named LPS1 and LPS5), Poly(I:C) at 20 and 40 µg/mL (groups named POLY20 and POLY40), or sham-medium condition for 2 or 24 h. In our study, Poly(I:C) and LPS were used as pathogen associated molecular patterns (PAMPs or positive controls) since Poly(I:C) is a viral mimetic and LPS is a bacterial endotoxin widely used in cell activation protocols with RAW and BV2 cells. At the end of the protocol, supernatant or cell extracts were collected. For MTT viability, nitrite, and cytokines assays, the cells were reseeded in 96-wells culture plates at 1x104 cells/well of density. In addition, the cells were reseeded in 24-wells culture plates at 1x105 cells/well for arginase activity assay. Finally, the cells were reseeded in 6-well culture plates containing a glass coverslip at 2x105 cells/well density for immunofluorescence assays. The tested concentrations of RBD peptide,^21^ LPS,^24^ and Poly(I:C)^25^ were based on previous *in vitro* studies of immune activation in these monocytic cell lineages.

*Cell viability test (MTT assay)* - The cells were seeded into 96-well plates for this assay and added a solution (10 μL) containing methyl tetrazolium (5 mg/mL MTT) to each well for 4 h at minimum. After this period, 100 µL of pure DMSO was added for cell lysis and formazan solubilisation. After 30 min shaking, the absorbance was measured in a microplate reader at 540 nm (Eon, Biotek Instruments, USA). Results were expressed as a percentage of untreated controls (considered as 100% of viability).

*Determination of nitrite levels* - Briefly, 150 μL of Griess Reagent (5% phosphoric acid, 1% sulfanilamide, 0.1% NEED) was added to 150 μL of the supernatant and incubated at room temperature for 10 min, protected from light. Absorbances were obtained at 540 nm in a microplate reader (Eon, Biotek Instruments, USA). Results were expressed as µM of nitrite/mL of culture medium.^26^

*Determination of arginase activity* - The arginase activity was determined as previously described.^27^ Total cell extracts were incubated with enzyme activation buffer (10 mM MnCl2, 50 mM Tris- HCl, pH 7.5) for 10 min at 55ºC and 50 µL of L-arginine solution - (0.5 M arginine, pH 9.7) for 70 min at 37ºC. To stop the reaction, we added 200 µL of buffer (H2SO4, H3PO4, and H2O 1:3:7). Finally, in a dark room, we added a 9% ISPF solution dissolved in absolute ethanol and heated at 100ºC for 45 min. The reaction product (200 µL) was read in a microplate reader at 540 nm (Eon, Biotek Instruments, USA). Arginase activity was expressed as µg of urea/µg of protein.

*Determination of cytokine production* - Culture supernatant was collected and used to determine the cytokines TNF, IL-6, and IL-10 levels. According to the manufacturer’s protocol, the cytokine concentration in 100 μL samples was determined by enzyme-linked immunosorbent assay (PeproTech, USA) and expressed in pg/mL of supernatant.

*Immunofluorescence staining for iNOS and cleaved-caspase-3* - Cells were fixed in 2.5% paraformaldehyde (PFA) in phosphate-buffered solution (PBS) for 25 min at 4ºC and subsequently washed and permeabilised in PBS buffer containing Tween 20 (0.02%) three times for 5 min. Next, the cells were incubated in 5% normal bovine serum in PBS containing Tween 20 overnight at 4ºC to attenuate nonspecific antibody binding. Then, cells were incubated overnight at 4ºC with antibodies targeting inducible nitric oxide synthase (iNOS) (1:200 rabbit monoclonal, Cell Signaling Technologies) or caspase 3 (1:200, caspase 3 - cleaved Asp175 Polyclonal Antibody, Thermo-Fisher Scientific). Subsequently, cells were incubated with the secondary antibody Alexa Fluor 568 donkey anti-rabbit IgG (1:400, Invitrogen, Karlsruhe, Germany) for 2 h. After this, hacks for both stainings were incubated with Lycopersicon esculentum (Tomato) Lectin- FITC conjugated for 2 h, gently washed three times with PBS, and then incubated with the nuclear dye DAPI (Invitrogen, Karlsruhe, Germany) for 15 min. Sections were then cover-slipped and examined. Images were acquired for at least six random fields using the ImageReader Cytation 3 (Biotek Instruments, USA) with a magnification of 10 and 20X. Quantification of the median fluorescence intensity (MFI) and the marked area was semi-automatically made using the FIJI-ImageJ software by a blinded experimenter.

*Nuclear morphometric analysis (NMA)* - Nuclear morphometric analysis was performed as previously described to screen cell fate (*i.e.*, apoptosis, senescence, or mitotic catastrophe) based on nuclear shape and size. Briefly, the cells were fixed with 4% paraformaldehyde and stained with DAPI 300 nM at room temperature in the dark. Images were acquired for at least six random fields using the ImageReader Cytation 3 (Biotek Instruments, USA) with a magnification of 20X. The nuclear contours were delimited using the magic wand tool, followed by the acquisition of the following variables: Area, Radius ratio (Rr), Roundness (Rou), Aspect (Asp), and Areabox (Arbx). After the acquisition, data were pasted into a spreadsheet available at www.ufrgs.br/labsinal/NMA, in which an analysis of nuclear area versus shape was performed. The nuclear shape is defined by the Nuclear Irregularity Index (NII), calculated using the formula: NII = Asp-Arbx+Rr + Rou. Through the NII analysis, nuclei are classified according to their size and shape in the following categories: normal (N), small and regular (SR), small and irregular (SI), large and regular (LR), and irregular (IR). N nuclei correspond to normal cells, SR nuclei correspond to apoptotic cells, LR and LIr correspond to nuclei from senescent cells, and SI and IR correspond to mitotic catastrophe.^28^ The results were expressed as the percentage of each cell category/total nuclei analysed in each field.

*Statistical analysis* - Statistical analysis and graphic construction were performed using GraphPad Prism 9 for macOS software, Version 9.3.1 (350), San Diego, California, USA. The results were compared by one-way analysis of variance (ANOVA). Normality was evaluated by Kolmogorov-Smirnov test. Tukey was used as a post hoc test when data assumed a Gaussian distribution. In contrast, Kruskal-Wallis’s test evaluated nonparametric data. Data are expressed as means ± standard error of the mean (SEM), and differences were considered statistically significant at p < 0.05. ANOVA results are present in figure legends.

## RESULTS

**Fig. 1:**
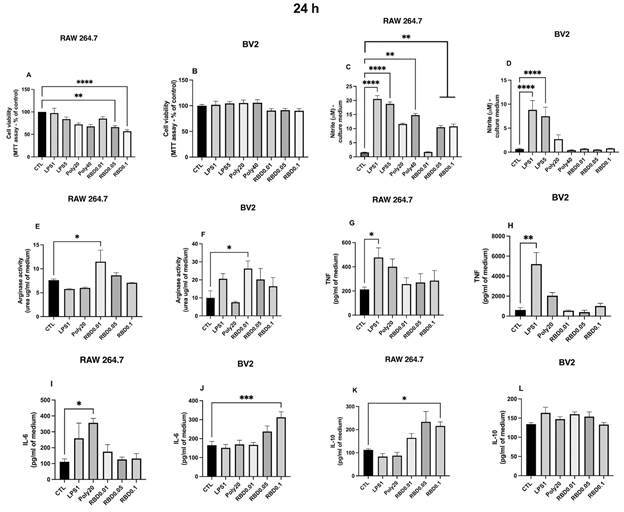
immune activation profile induced by severe acute respiratory syndrome coronavirus 2 (SARS-CoV-2) S1 receptor-binding domain (RBD) protein exposure for 24 h to RAW264.7 macrophages and BV2 microglia cells. Macrophages or microglial cells were exposed to RBD (0.01, 0.05, and 0.1 µg/mL), LPS (1 or 5 µg/mL), or POLY I:C (20 or 40 µg/mL) for 24 h and supernatants and cell extracts samples were collected. Panels represent (A and B) Cell viability (MTT assay) in % of controls, (C and D) nitrite concentrations in culture supernatant, (E and F) arginase activity in total cell extracts, (G and H) TNF, (I and J) IL-6 and (K and L) IL-10 concentrations in culture supernatant. Bars represent the mean ± standard error of the mean (SEM). The data presented are from three independent experiments. Data were analysed using one-way analysis of variance (ANOVA) followed by Tukey or Kruskal-Wallis tests. CTL: control; LPS: lipopolysaccharide; POLY I:C: polyinosinic:polycytidylic acid; MTT: 3-[4,5-dimethylthiazol-2-yl]-2,5 diphenyl tetrazolium bromide; TNF: tumour necrosis factor alpha; IL: interleukin.

**Fig. 2:**
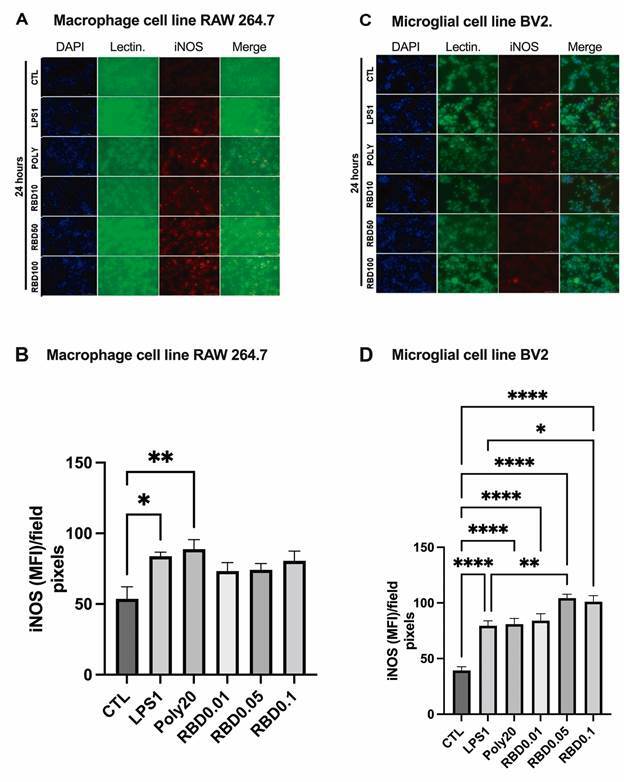
iNOS (red) and Tomato-lectin (green) staining in macrophage cell line RAW 264.7 (A,B) and microglial cell line BV2 (C,D). DAPI (blue) was used as a nuclear marker. The slides were immunostained with anti-iNOS antibody and FITC- conjugated tomato-lectin and examined with a Cytation3 fluorescence microscope (magnification 20×). The cells were exposed to saline, receptor-binding domain (RBD) (0.01, 0.05, and 0.1 µg/mL), LPS (1 µg/mL), or POLY I:C (20 µg/mL) for 24 h. The data presented are from three independent experiments. CTL: control; LPS: lipopolysaccharide; POLY I: C: polyinosinic: polycytidylic acid; iNOS: inducible oxide nitric synthetase.

*Recombinant RBD peptide stimulation induced cell activation and cytotoxicity in RAW 264.7 macrophages and BV2 microglial cells* - After 24 h exposure of RAW 264.7 cells to the PAMPs (Fig. 1A), we detected a significant decrease in cell viability only after RBD0.05 (p = 0.0022) and RBD0.1 (p < 0.0001) stimulation compared with control conditions (Fig. 2A). On the other hand, we did not observe cell viability alterations in BV2 cells (Fig. 1B). Nitrite levels were significantly higher in RAW264.7 cells exposed to RBD 0.05 and 0.1 (p < 0.01) as well as to the positive controls, LPS1 and 5 (p < 0.0001) and POLY40 (p < 0.01) compared with control conditions (Fig. 1C). Nitrite levels were unaltered in BV2 cells exposed to RBD. Still, in BV2 cells, the positive control LPS, in both concentrations, significantly increased nitrite content compared with control conditions (p < 0.0001) (Fig. 1D). Regarding arginase activity (Fig. 1E-F), it was increased in RAW264.7 (p = 0.0406) and BV2 (p < 0.05) cells stimulated with RBD0.01 compared with control. TNF levels were not influenced by RBD exposure in RAW264.7 and BV2 cells (Fig. 1G-H) but were higher after LPS1 exposure (p < 0.05). The levels of IL-6 (Fig. 1 I-J) were higher in BV2 cells exposed to RBD0.1 (p = 0.0007). In contrast, in RAW 264.7 cells, only the positive control, POLY20 (p = 0.0419), increased the levels of this cytokine compared with the control. Stimulation with RBD0.1 also increased IL-10 levels in RAW264.7 cells compared with the control (Fig. 1K). No significant alterations were detected in IL-10 levels in BV2-stimulated cells (Fig. 1L).

We observed minor changes by 2 h exposure of RAW 264.7 and BV2 cells to the PAMPs [Supplementary data (Fig. 1)].

*iNOS immunostaining increased after RBD exposure after 24 h in BV2 cells but not in RAW 264.7 cells* - iNOS staining significantly increased in BV2 cells exposed to all concentrations of RBD, *i.e.*, RBD0.01 (p < 0.0001), RBD0.05 (p < 0.0001) and RBD0.1 (p < 0.0001), as well as to the positive controls LPS1 (p < 0.0001), and POLY20 (p < 0.0001) compared with control conditions (Fig. 2C-D). It should be mentioned that iNOS expression was higher in the cells exposed to RBD0.05 (p = 0.0051) and 0.1 (p = 0.0253) than in the cells stimulated with LPS1. Regarding RAW 264.7 cells (Fig. 2A-B), only the positive controls LPS1 (p = 0.0114) and POLY20 (p = 0.0017) increased iNOS expression compared with control conditions. On the other hand, 2 h after cells’ exposure to the PAMPs [Supplementary data (Fig. 2A-B, E-F)], we only observed increased iNOS expression in BV2 cells exposed to LPS1 compared with control (p = 0.0442).

**Fig. 3:**
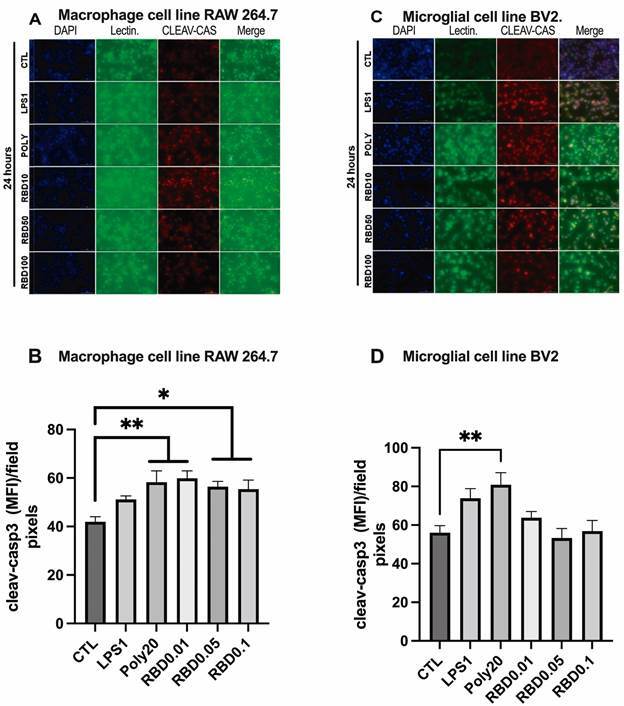
cleaved-caspase 3 (red) and Tomato-lectin (green) staining in macrophage cell line RAW 264.7 (A,B) and microglial cell line BV2 (C,D). DAPI (blue) was used as a nuclear marker. The slides were immunostained with anti-cleaved caspase-3 antibody and FITC- conjugated tomato-lectin and examined with a Cytation3 fluorescence microscope (magnification 20×). The cells were exposed to saline, receptor-binding domain (RBD) (0.01, 0.05, and 0.1 µg/mL), LPS (1 µg/mL), or POLY I:C (20 µg/mL) for 24 h. The data presented are from three independent experiments. CTL: control; LPS: lipopolysaccharide; POLY I: C: polyinosinic:polycytidylic acid.

*Cleaved caspase-3 immunostaining was higher only in RAW 264.7 cells after RBD exposure but not in BV2 microglia cells* - Due to the observed alterations in cell viability after 24 h stimulation with RBD peptide, we decided to evaluate the expression of cleaved caspase-3 as a marker of apoptosis. In RAW 264.7 cells (Fig. 3A-B), all concentrations of RBD [RBD0.01 (p = 0.0023), RBD0.05 (p = 0.0128), and RBD0.1 (p = 0.0113)] as well as POLY20 (p = 0.0020) significantly increased cleaved caspase-3 staining compared with control. On the other hand, only POLY20 significantly increased this marker in BV2 cells compared with the control (p < 0.01) (Fig. 3C-D).

After 2 h exposure [Supplementary data (Fig. 2-D, G-H)], RBD0.1 significantly increased cleaved caspase 3 expression compared with control conditions, RBD0.01, RBD0.05, LPS, and POLY (p < 0.0001). Regarding BV2 cells, only LPS1 increased cleaved caspase 3 expression compared with control conditions (p < 0.01).

**Fig. 4:**
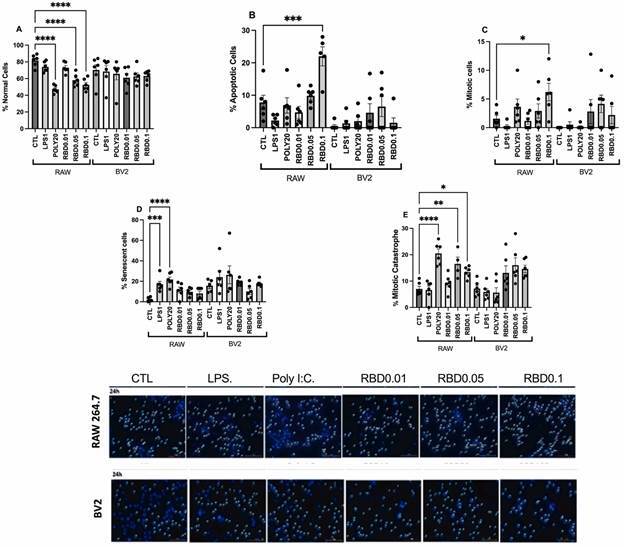
panel of nuclear morphometric analysis (NMA). The slides were stained with the nuclear marker DAPI (blue) and examined with a Cytation3 fluorescence microscope (magnification 20×). A: % of normal cells; B: % of apoptotic cells; C: % of mitotic cells; D: % senescent cells; E: % mitotic catastrophe in RAW and BV2 cells at CTL, RBD (0.01, 0.05 and 0.1 µg/mL), LPS (1 µg/mL) or poly I: C (20 µg/mL)-treated conditions at 24 h post exposition. F: representative panel. The data presented are from three independent experiments, and at least six random fields of each group were included. CTL: control; RBD: receptor binding domain protein; LPS: lipopolysaccharide; poly I:C: polyinosinic:polycytidylic acid.

*RBD peptide stimulation changes the cell fate after exposure* - The nuclear morphometric analysis evaluated the cell fate (morphology, apoptosis, mitosis, and senescence) under stimulation (Fig. 4). After a 24 h exposure to the PAMPs, RAW264.7 cells presented a significant reduction in the % of normal morphology when exposed to RBD0.05, RBD0.1, and POLY20, compared with control (p < 0.0001), which was not observed in BV2 cells (Fig. 4A). Furthermore, in RAW264.7 but not BV2 cells, the % of apoptotic nuclei significantly increased after RBD0.1 exposure (Fig. 4B) compared with control (p = 0.0010). Moreover, we observed an increased % of RAW264.7 cells presenting mitotic morphology after RBD0.1 stimulation but not BV2 cells (Fig. 4C). Regarding senescent cells, only the positive controls LPS1 and POLY20 caused a significant increase in this parameter in RAW264.7 cells but not in BV2 cells (Fig. 4D) compared with control (p < 0.0001). Finally, evaluating the % mitotic catastrophe morphology, we found that stimulation with RBD0.05, RBD0.1, as well as POLY20 significantly increased this parameter in RAW264.7 cells compared with control conditions (p = 0.0011) (Fig. 4E).

As depicted in Supplementary data (Fig. 3), the 2 h exposure of RAW 264.7 cells to RBD0.1 increased the % of senescent cells compared with control conditions. On the other hand, RBD exposure to BV2 cells caused: decreased % of normal cells with RBD0.1, an increased % of mitotic cells with RBD0.05, and an increased % of mitotic catastrophe with RBD0.01 and 0.05 compared with control.

## DISCUSSION

Here, we showed, as far as we know for the first time, that the RBD exposure at the highest concentration for 24 h has main immunogenic and pro-inflammatory effects mainly in microglial cell lines with M1 predominant features such as increased iNOS expression and IL-6 secretion. This effect was more pronounced with RBD than the other PAMPs since LPS was used in concentrations 10-100 times higher than RBD, while Poly(I:C) was 200 to 2000 times. On the other hand, we observed in RAW264.7 cells an M2b alternative activation phenotype (characterised by increased arginase and IL-10 levels) and a higher expression of cleaved caspase 3, revealing activation of apoptotic mechanisms. Since most previous studies were centred on the effects of the glycoprotein S1, we add further knowledge about the immunogenic profile of the RBD peptide in macrophage and microglial cells. Therefore, our results contribute to understanding the immunopathology and neuropathology of SARS-CoV-2 infection and the possible future development of vaccines based on this peptide.

Monocytes and macrophages have been hypothesised as cytokine releases. These cells are the primary triggers and keepers of the cytokine storm syndrome.^29^ Indeed, previous clinical reports have described significant associations between altered monocyte-macrophage markers, such as count, phenotype, complexity, and macrophage invasion and exudation, with clinical variables of severe infection, such as liver failure, pancytopenia, severe hypoxemia, and acute myocardial injury.^30^ ^,^ ^31^ ^,^ ^32^ Similarly, in the CNS, previous studies have reported increased leptomeningeal inflammation, macrophagic invasion, blood-brain barrier damage, and increased microglial activation and cluster formation in post-mortem brain samples of severe COVID-19.^33^ ^,^ ^34^

We used murine cell lineages; hence they do not present human ACE2 protein. Despite this, recent evidence points to the novel variants of this virus as capable of infecting common mice, such as BALB/c and C57BL/6, inducing significant pathological lung lesions and inflammatory responses.^18^ Furthermore, although previous predictive analysis has suggested that ACE2 gene-encoded proteins are highly homologous across mammals,^35^ recent reports have demonstrated that the S1 protein mediates a clear pro-inflammatory activation state in several macrophagic cell lineages and primary cultures through direct interaction and activation of other receptors, such as TLR4 and TLR2,^19^ ^,^ ^36^ being considered a PAMP.^37^ Indeed, the S protein, but not M, N, and E proteins revealed to be a potent viral PAMP, stimulating macrophages, monocytes, and lung epithelial cells.^37^ In line with this, our results showed that the exposure of macrophages to the isolated RBD domain promotes a mixed anti- and pro-inflammatory state, characteristic of TLR4 ligands. Indeed, TLR4 activation may induce the synthesis of pro-inflammatory cytokines and anti-inflammatory ones, such as IL-10, which depends on ERK1/2 and p38 MAPK activation.^38^ However, despite being a promising mechanism, we did not demonstrate a causal association between ERK1/2 and p38 MAPK activation mediated by TLR4 and RBD pro-/anti-inflammatory cytokine profile, which remains an important research question.

Still, in macrophages, we observed a cytotoxic effect with increasing concentrations of RBD. Programmed cell death is a host defence mechanism triggered by cell activation, stress, or damage. RBD markedly increased apoptotic nuclei in RAW264.7 cells differently from Poly(I:C). At the same time, Poly(I:C) caused a senescent and mitotic catastrophe nuclei phenotype. We did not detect any significant cytotoxic effect of the RBD in BV2 microglial cells as previously described. However, the S1 protein induced in human microglial cells increased the expression of pro-inflammatory cytokines, disrupted mitochondria biogenesis, increased ROS production, and augmented apoptosis rate.^20^ Instead, we detected an M1 activation profile with a marked increase in iNOS expression. Excessive NO synthesis under neuroinflammation leads to the formation of reactive nitrogen species and neuronal cell death, associated with excitotoxicity processes induced by glutamate accumulation and microglial activation, contributing to the increased age-associated susceptibility to neurodegenerative disorders.^39^ Therefore, this mechanism may partially explain the neuropsychiatric complications of COVID-19.

The differences observed here in the immune activation profile and RBD’s cytotoxic effects between RAW264.7 and BV2 cell lineages could be attributed to their distinct embryonic origin and the pattern of expression and responsivity of most PRR. Although microglia and macrophages require the transcription factor Pu.1,^40^ the latter necessitates Myb and FLT3.^41^ In contrast, microglial development is csf1-receptor-dependent and FLT3- and Myb-independent.^42^ ^,^ ^43^ ^,^ ^44^ Thus, these two myeloid populations have a unique set of transcription factors leading to a diverse gene expression pattern and immune responsivity, as demonstrated in models for virus-induced neuroinflammation.^45^

Although previous evidence shows the S1 ability to bind and activate TLRs, additional targets, such as CD147 and the transmembrane protease serine 2 (TMPRSS2), have been described for virus-host cells interaction.^46^ Interestingly, some of these targets have their expression highly variable between tissues and organs, which can afford different mechanisms of virus entry according to the tissue site.^47^ Indeed, CD147 is a transmembrane glycoprotein of the immunoglobulin superfamily and contributes to intercellular recognition.^48^ Furthermore, a recent study demonstrated an increased CD147 expression in mice brain tissue and neuronal and microglia (BV2) cell lineages compared to ACE2 and TMPRSS2.^49^ Therefore, CD147 could be an additional potential target for S1 and potentially for the RBD domain, contributing to the virus’s neuropsychiatric manifestations.

The present study has some limitations since we did not use cell state-specific marker genes for evaluating cell fate.

*In conclusion* - We showed that SARS-CoV2 S1-RBD induces a distinct and dose-dependent immune activation profile in macrophage and microglial cell lineages. Indeed, our results demonstrated that the highest concentrations of RBD promoted a powerful cytotoxic pro-apoptotic effect in RAW264.7 and M2b alternative activation profile, characterised by the increased production of nitrite, IL-10, and arginase accompanied by changes in cell morphology.

On the other hand, at the highest concentration, RBD induced an M1 activation profile in BV2 microglial cells, causing increased IL-6 production and a strong iNOS expression, which could be the underlying mechanism of the virus-induced neuropsychiatric alterations. At the lowest concentration, RBD peptide induced arginase activity in both cell lines, revealing an immunoregulatory phenotype.

As a perspective, future studies must be designed to study the immunogenic profile of RBD obtained from the current variants.
