# Supplementary material for: In vitro immunogenic profile of recombinant SARS-CoV2 S1-RBD peptide in murine macrophage and microglial cells
Source: Mem Inst Oswaldo Cruz. 2023 Mar 31;118:e220144. doi: 10.1590/0074-02760220144 (PMC10065410; doi:10.1590/0074-02760220144)
Supplement: Supplementary file 1 [file 1678-8060-mioc-118-e220144-s.pdf]

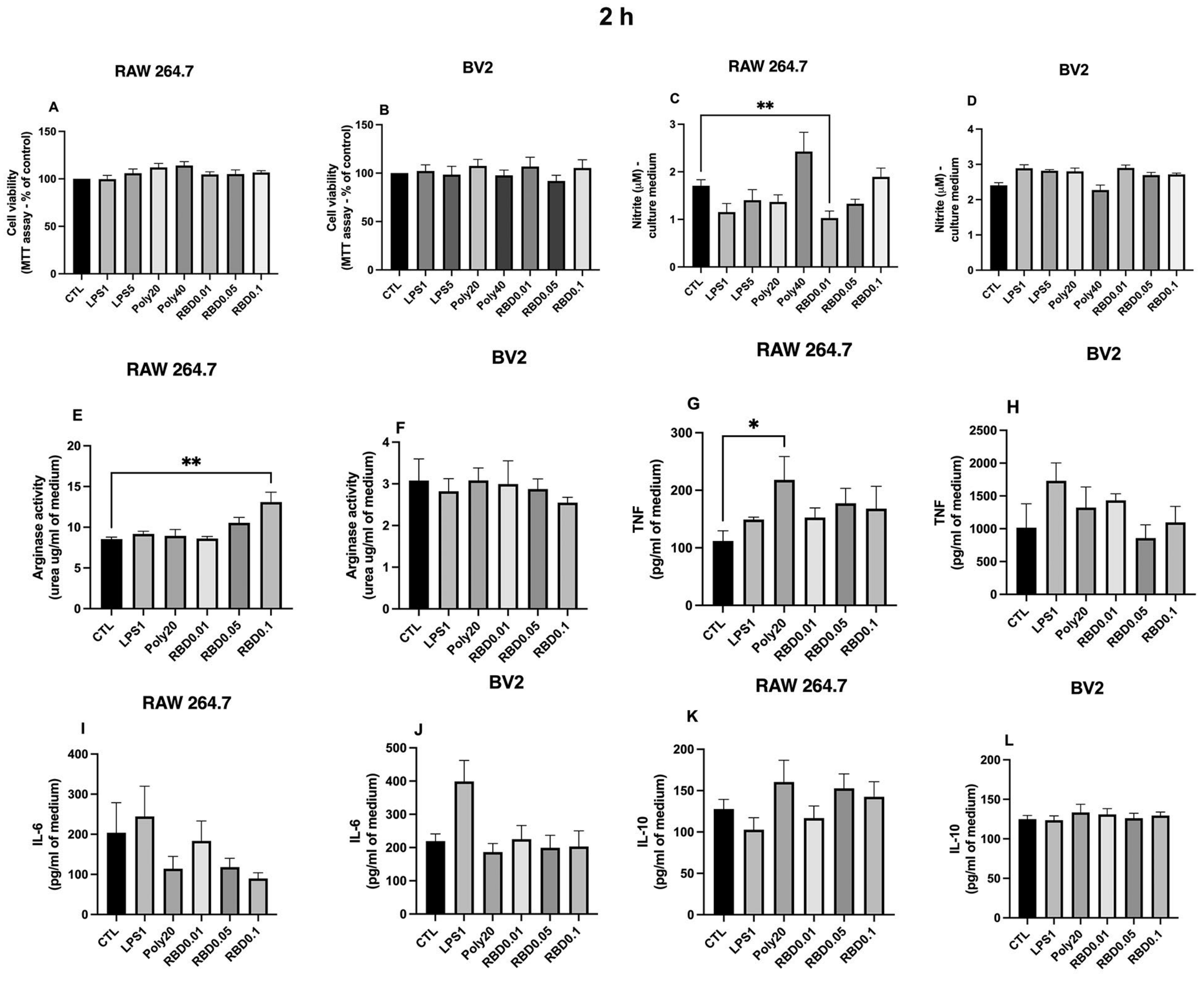

Fig. 1: immune activation profile induced by severe acute respiratory syndrome coronavirus 2 (SARS-CoV-2) S1 receptor-binding domain (RBD) protein exposure for 2 h to RAW264.7 macrophages and BV2 microglia cells. Macrophages or microglial cells were exposed to RBD (0.01, 0.05, and 0.1 μg/mL), lipopolysaccharide (LPS) (1 or 5 μg/mL), or POLY I:C (20 or 40 μg/mL) for 2 h and supernatants and cell extracts samples were collected. Panels represent (A and B) Cell viability (MTT assay) in % of controls, (C and D) nitrite concentrations in the culture supernatant, (E and F) arginase activity in total cell extracts, (G and H) TNF, (I and J) IL-6 and (K and L) IL-10 concentrations in the culture supernatant. Bars represent the mean ± standard error of the mean (SEM). The data presented are from three independent experiments. Data were analysed using one-way analysis of variance (ANOVA) followed by Tukey or Kruskal-Wallis tests. *$p < 0.05$, **$p < 0.01$, ***$p < 0.001$, ****$p < 0.0001$. CTL: control; POLY I:C- polyinosinic:polycytidylic acid; MTT: 3-[4,5-dimethylthiazol-2-yl]-2,5 diphenyl tetrazolium bromide; TNF: tumour necrosis factor alpha; IL: interleukin.

CC BY

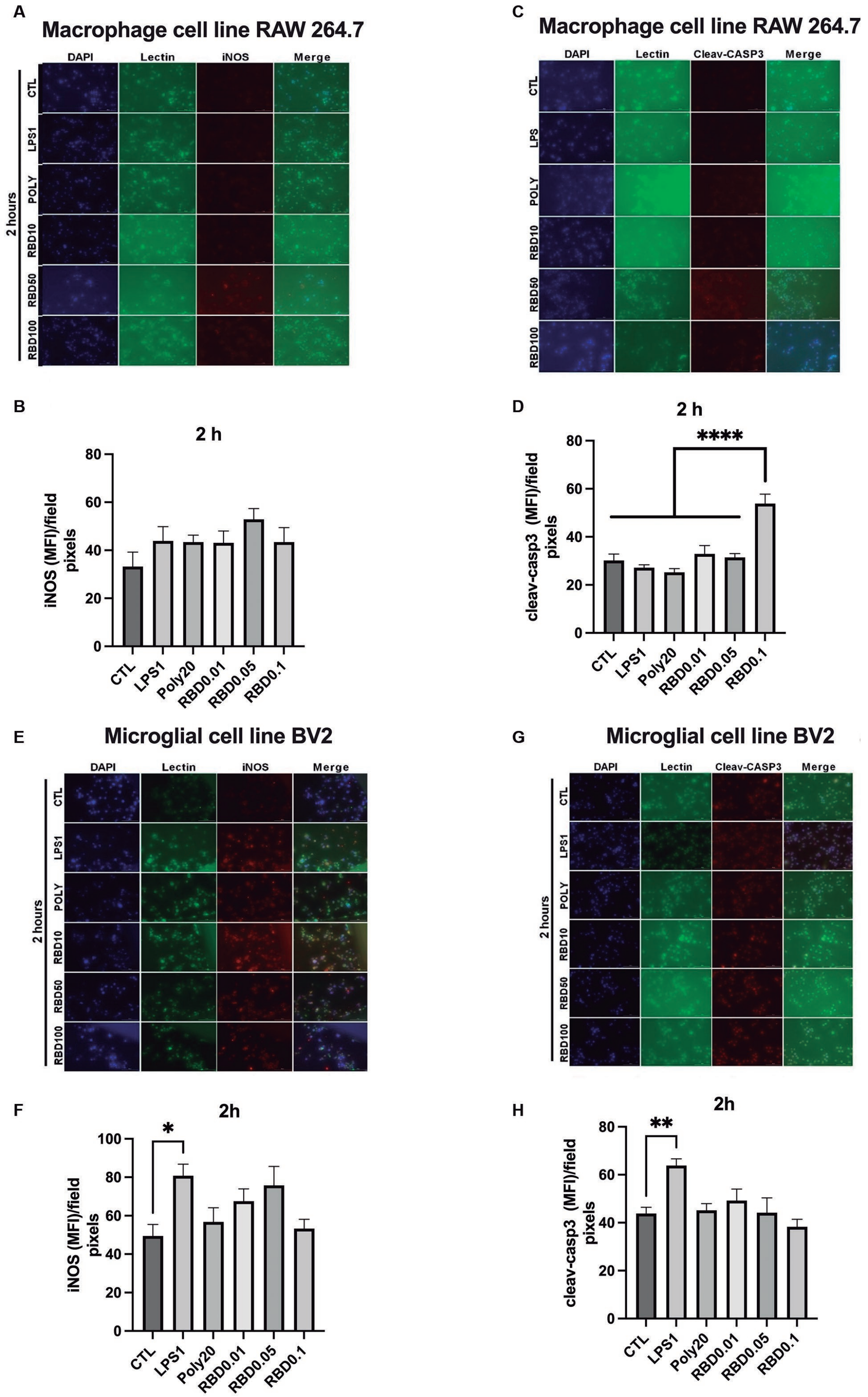

Fig. 2: iNOS (red) and Tomato-lectin (green) (A,B,E,F) and cleaved-caspase 3 (red) and Tomato-lectin (green) (C,D,G,H) staining in macrophage cell line RAW 264.7 and microglial cell line BV2. DAPI (blue) was used as a nuclear marker. The slides were immunostained with anti-iNOS antibody and FITC-conjugated tomato-lectin or anti-cleaved caspase-3 antibody and examined with a Cytation3 fluorescence microscope (magnification 20×). The cells were exposed to saline, receptor binding domain protein (RBD) (0.01, 0.05, and 0.1 μg/mL), lipopolysaccharide (LPS) (1 μg/mL), or POLY I:C (20 μg/mL) for 2 h. The data presented are from three independent experiments. CTL: control; POLY I: C: polyinosinic:polycytidylic acid; iNOS: inducible oxide nitric synthetase.

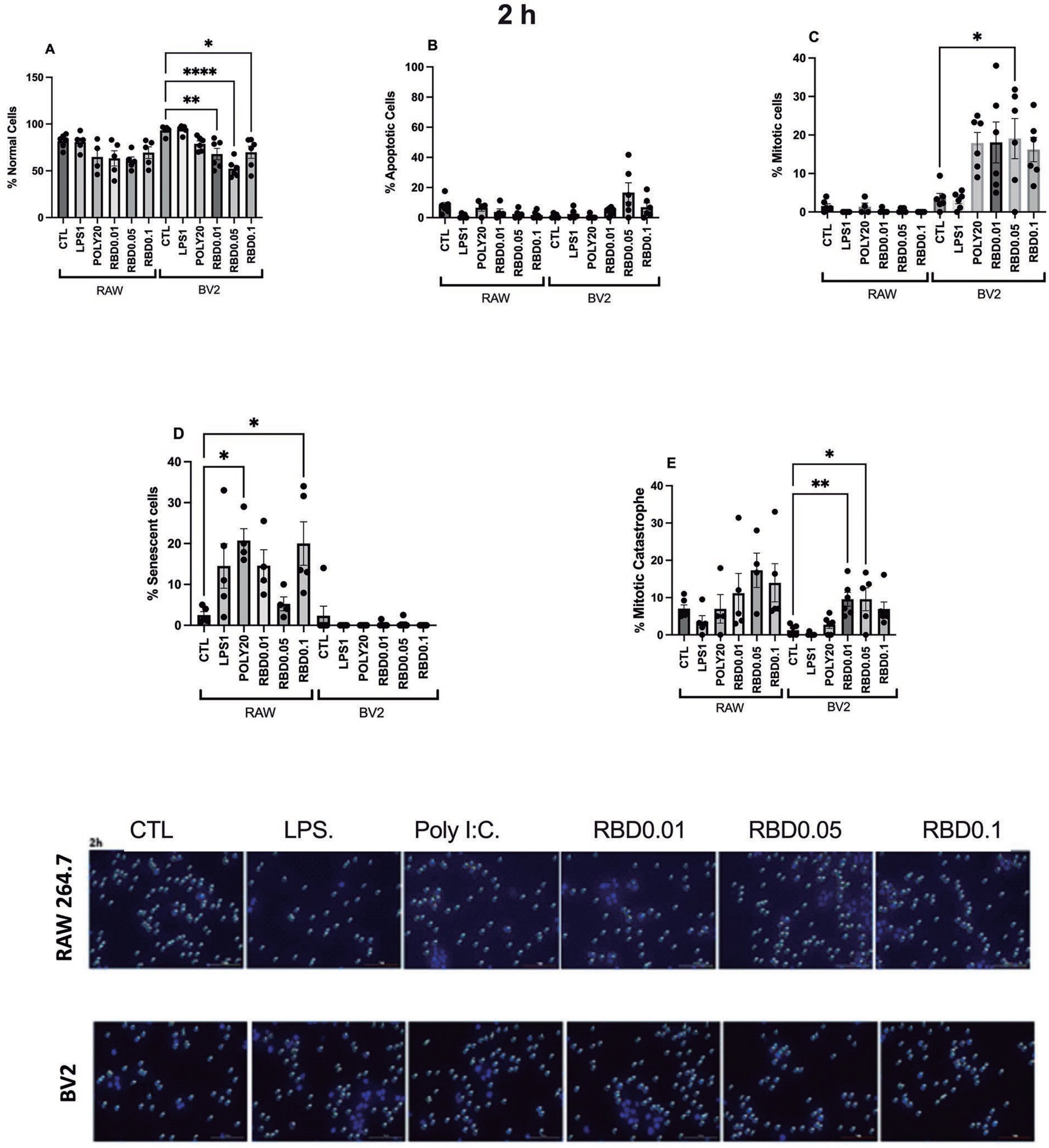

Fig. 3: panel of nuclear morphometric analysis (NMA). The slides were stained with the nuclear marker DAPI (blue) and examined with a Cytation3 fluorescence microscope (magnification 20×). A: % of normal cells; B: % of apoptotic cells; C: % of mitotic cells; D: % senescent cells; E: % mitotic catastrophe in RAW and BV2 cells at CTL, receptor binding domain protein (RBD) (0.01, 0.05 and 0.1 µg/mL), lipopolysaccharide (LPS) (1 µg/mL) or poly I: C (20 µg/mL)-treated conditions at 2 h post exposition. K: representative panel. The data presented are from three independent experiments; at least six random fields of each group were included. CTL: control; poly I:C: polyinosinic:polycytidylic acid.
